# Movements of scalloped hammerhead sharks (*Sphyrna lewini*) at Cocos Island, Costa Rica and between oceanic islands in the Eastern Tropical Pacific

**DOI:** 10.1371/journal.pone.0213741

**Published:** 2019-03-12

**Authors:** Elena Nalesso, Alex Hearn, Oscar Sosa-Nishizaki, Todd Steiner, Alex Antoniou, Andrew Reid, Sandra Bessudo, Germán Soler, A. Peter Klimley, Frida Lara, James T. Ketchum, Randall Arauz

**Affiliations:** 1 Department of Biological Oceanography, Centro de Investigación Científica y de Educación Superior de Ensenada, Ensenada, Baja California, México; 2 Programa Restauración de Tortugas Marinas, San José, Costa Rica; 3 Colegio de Ciencias Biológicas y Ambientales / Galapagos Science Center, Universidad San Francisco de Quito, Quito, Ecuador; 4 Turtle Island Restoration Network, Forest Knolls, California, United States of America; 5 MigraMar, Forest Knolls, California, United States of America; 6 Fins Attached Marine Research and Conservation, Colorado Springs, Colorado, United States of America; 7 Jurassic Shark Expeditions, Dorchester, United Kingdom; 8 Fundación Malpelo y Otros Ecosistemas Marinos, Bogotá, Colombia; 9 Institute of Marine and Antarctic Studies, University of Tasmania, Tasmania, Australia; 10 Department of Wildlife, Fish & Conservation Biology, University of California, Davis, California, United States of America; 11 Pelagios Kakunjá A.C., La Paz, Baja California Sur, México; Havforskningsinstituttet, NORWAY

## Abstract

Many species of sharks form aggregations around oceanic islands, yet their levels of residency and their site specificity around these islands may vary. In some cases, the waters around oceanic islands have been designated as marine protected areas, yet the conservation value for threatened shark species will depend greatly on how much time they spend within these protected waters. Eighty-four scalloped hammerhead sharks (*Sphyrna lewini* Griffith & Smith), were tagged with acoustic transmitters at Cocos Island between 2005–2013. The average residence index, expressed as a proportion of days present in our receiver array at the island over the entire monitoring period, was 0.52±0.31, implying that overall the sharks are strongly associated with the island. Residency was significantly greater at Alcyone, a shallow seamount located 3.6 km offshore from the main island, than at the other sites. Timing of presence at the receiver locations was mostly during daytime hours. Although only a single individual from Cocos was detected on a region-wide array, nine hammerheads tagged at Galapagos and Malpelo travelled to Cocos. The hammerheads tagged at Cocos were more resident than those visiting from elsewhere, suggesting that the Galapagos and Malpelo populations may use Cocos as a navigational waypoint or stopover during seasonal migrations to the coastal Central and South America. Our study demonstrates the importance of oceanic islands for this species, and shows that they may form a network of hotspots in the Eastern Tropical Pacific.

## Introduction

Oceanic islands and seamounts provide important habitats in the pelagic environment for many marine species, often resulting in biological “hotspots” characterized by a greater diversity and abundance of pelagic life [1]. They can be thought of as “border environments” where reef-associated communities interact with a suite of open water species of different trophic levels, from planktivorous fishes to top predators [2]. Sharks in particular, often aggregate at oceanic islands. However, these areas may not necessarily be, energetically speaking, their most important habitat [3]. While these locations may provide refuge, cleaning, or navigation reference points [4–9], sharks may be particularly vulnerable here to fishing gear targeting their aggregations.

Currently, one quarter of all shark and ray species are threatened according to the IUCN Red List criteria due to overfishing (both targeted and incidental) [10]. In particular, the scalloped hammerhead shark (*Sphyrna lewini* Griffith & Smith), is of special concern. *S*. *lewini* is listed as Endangered by the IUCN Red List of Endangered Species [11] due to reduction in population sizes based on studies over multiple ocean basins [12–14]. This coastal-pelagic species is found in temperate and tropical oceans throughout the planet [15], and is known to form big aggregations around oceanic islands [15–19].

Marine Protected Areas (MPAs) are one of many tools used to protect both commercial and non-commercial marine species [20–22]. The Eastern Tropical Pacific (ETP) contains a network of MPAs surrounding the main oceanic islands and some coastal areas, among which Cocos Island MPA (Costa Rica) is of particular importance as it is home to more than half the endemic species of this region [2]. At Cocos and at neighbouring oceanic islands (Malpelo, Colombia; Galapagos, Ecuador and Revillagigedo, Mexico), large, female-dominated aggregations of adult scalloped hammerhead sharks are common at certain times of the year ([17,18,19,23], Ketchum personal communication). Although males are present in these aggregations, these appear to be more solitary and more common in open waters [24,25]. However, juvenile and neonate scalloped hammerhead sharks are rare at these oceanic locations and it is thought that pupping grounds for these populations occur along mainland of Central and South America [26–29].

Sharks have shown a particularly positive response to the recovery of their populations in some MPAs, such as in Fernando de Noronha, Brazil [30]. However, at both Cocos and Malpelo, the creation of MPAs does not appear to have halted a declining trend in the abundance of scalloped hammerheads sharks [23,31,32]. This is thought to be the combined result of intense targeted fishing pressure on sharks outside the 22 km protected area, but is also attributed to illegal fishing within the MPA [32,33]. Although longline fishing effort in the Cocos region targets tuna, sharks constitute a main component of the catch and are often retained as marketable by-catch due to the high value of their fins [34].

The objective of this paper is to determine the residency and site preference of scalloped hammerhead sharks at Cocos Island National Park MPA. We define residency as the number of days during which a shark is detected at least once at the island as a proportion of the track length, from the date of tagging to the last detection. However, given that our coverage of the island is limited to six locations, in reality this is a minimum residence time. Site preference is defined as the relative proportion of time spent at each study site area, assuming that their detection ranges remain comparable throughout the study period. We also explore the occurrence of long-range movements to other MPAs in the region, as a key aspect in improving understanding of the hammerhead shark movement ecology, and for designing effective conservation [35]. We used Network Analysis (NA) to identify frequent movements within core use areas, highlighting important movement corridors between locations [36]. Network Analysis provides greater insight into the importance and connectivity of specific habitat features on the animal moving between them. It also proves valuable in revealing important information on distinct spatial and temporal changes in animal movement [37].

## Materials and methods

### Ethics statement

The work carried out in this study was done so in accordance with the following research permits (resolutions) from the Cocos Island National Park Authorities: 002-004-2004, 2007-ACMIC-008, 2008-ACMIC-012, 2009-ACMIC-006 2010-I-ACMIC-006, 2011-ACMIC-001, 2012-IACMIC006, ACMIC-I-2013-0012, ACMIC-I-2014-0007, ACMIC-I-2014-0015, ACMIC-I-2015-008 and 2016-I-ACMIC-07. Research methods for this study were approved by the Institutional Animal Care and Use Committee Protocol #16022, issued to the three co-authors based at the University of California, Davis at the time of the study (APK, AH & JTK).

### Study site

**Fig 1:**
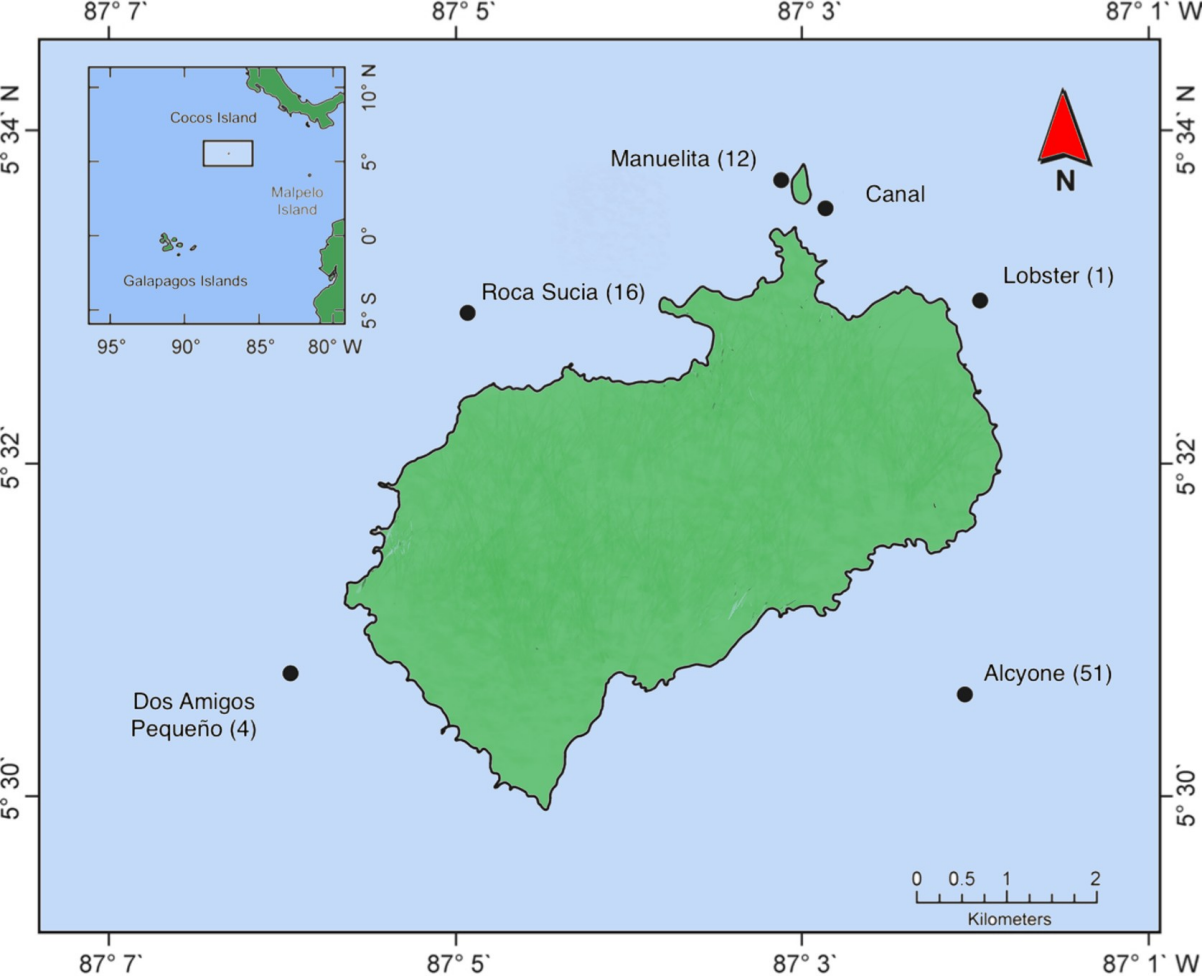
Location of Cocos Island and receiver array. Black dots represent the location of the receivers around the island. In parentheses, the number of sharks tagged in each site. The inset box indicates the location of the island in the Eastern Tropical Pacific.

Cocos Island (Costa Rica) is a small (23 km^2^) oceanic island in the ETP, which was declared a National Park in 1978 [38] and a UNESCO World Heritage Marine Site in 1997. The island lies 550 km to the southwest of the coast of Costa Rica (5°30’-5°34’ N, 87°01’-87°06’ W), approximately 710 km northeast of the Galapagos Islands and 627 km northwest of Malpelo (Fig 1), in an area of trade wind convergence that brings high rainfall to the island [39]. The mean sea surface temperature ranges from 26.8°C in the coldest months (October to December) to 28.4°C in the warmest months (April through June) [40]. The island is influenced by the North Equatorial Counter-Current (NECC), which is at its strongest from August to September [40,41]. The coastline is mostly rocky and steep, with a narrow platform to the northwest, and a wide, less pronounced slope to the southeast [42–44].

### Acoustic telemetry

Nineteen research trips were carried out to Cocos from July 2005 to November 2013, during which coded, ultrasonic tags (V16, VEMCO Ltd, Halifax, Canada; diameter of 16 mm, length of 68 mm, 158 dB power output, 1350 days battery life and pseudorandom delay of 60–90 seconds to avoid repeated signal collision) were attached on 84 scalloped hammerhead sharks (S1 Table). The tags were tethered to stainless steel darts, which were inserted into the muscle of the shark at the base of the dorsal fin by SCUBA divers using pole spears or spear guns. Where possible, divers recorded the gender (determined by the presence or absence of claspers). The hammerhead sharks around Cocos Island mostly appeared to be in the sub-adult and adult size range (150–250 cm total length). Given the errors associated with multiple divers estimating in-water length of the hammerheads, we did not formally estimate the length of the sharks, and none of the divers reported tagging particularly large or small individuals.

Six underwater receivers (VR2W, VEMCO Ltd, Halifax, Canada) were deployed at different locations and depths: Alcyone (32 m), Roca Sucia (32 m), Dos Amigos Pequeño (32 m), Manuelita (39 m), Canal (24 m) and Lobster (24 m) (Fig 1). Each receiver was attached to a 3 m rope or cable tethered to a concrete base and a sub-surface buoy. These receivers can detect the pulses emitted by the coded tags at a nominal distance of 500 m according to the manufacturer (www.vemco.com), although in range tests carried out at Cocos, tag detection dropped off steeply after 150 m, and tags were not detected at distances greater than 300 m. These results were consistent with range test results at nearby Malpelo and Galapagos, where the effective detection range was limited to less than 250 m [17,18]. The data record for each receiver (tag ID, date and time) was downloaded every 6–9 months.

### Data analysis

Time series of presence/absence of each shark at Cocos Island were created from the detection records from all receivers. Where possible, we included information from receivers deployed at other locations in the ETP, made available through the regional MigraMar receiver network (www.migramar.org), which includes receivers located in the Galapagos Islands and Malpelo Island. Similarly, we included data from sharks tagged at other locations that were detected on our receiver array at Cocos.

An overall residency index (RI) was calculated based on [45] and [46], who defined RI as the number of days on which an individual was detected at the island divided by the total tracked number of days, from the date on which the shark was tagged to the date of its last detection. Thus, RI = 1 when the shark is detected at the island on each day from the moment of tagging to its last detection, and approaches 0 as the number of days detected in relation to the total track length decreases. It is important to mention that because the array does not cover the entire island, this value should be considered conservative, as sharks may be present at the island without being detected on the array.

In addition to the overall RI, we calculated number of visits and visit duration at each receiver for 17 sharks: 14 tagged at Alcyone, 2 at Roca Sucia and 1 at Manuelita (S1 Table), all of which were being monitored within the period of September 2011 to November 2013. These sharks were chosen because many of the other track lengths were short (<1 month) and this was the longest continuous period without gaps in receiver data due to battery failure or receiver loss (S1 Fig). The number of visits to each of the six receiver locations was estimated following the method outlined by [19], which considered that a shark swimming at an average speed of 0.5 ms^-1^ would take approximately 15 minutes to cross the entire detection range of a receiver. Thus for any shark displaying a string of detections shorter than 15 minutes at a particular location, we assumed that the shark was passing through the area rather than residing at that location. We used the V-track package in R ([47], R Development Team 2011), which is designed specifically to analyse VEMCO detections from receiver arrays, by grouping them into residence and non-residence events using user-defined criteria of thresholds to define the minimum number of successive pings (in this case: 2) detected at a receiver before a residence event is recorded, and the minimum time period in seconds between pings before a residence event is recorded (in this case: 900 seconds). A residency event was also considered to have terminated if the tag was detected twice or more at a different receiver. The number and duration of residence events were then compared between locations. To account for potential tagging bias (14 of the 17 sharks were tagged at Alcyone) we disregarded the first visit at the tagging location (because we do not know how long the shark was at the site before it was tagged), and then disregarded the subsequent portion of each track, such that for each shark, the utilized track began either a) when the shark was first detected at a site other than that where it was tagged or b) after the shark had been absent for at least 24 hours from its tagging location. The corrected dataset included 2034 visits, ranging from 26 (Canal) to 835 (Alcyone) and a total time spent over all sites of 1400.9 hours. We then used a linear mixed model using the “lmer” command of the R package lme4 [48,49]. In this case, the simple model estimates the time spent across all sharks but allows the average time spent to vary between sharks. We used a likelihood approach to compare a null model with models using “location” as a fixed effect and “individual fish (or Tag ID)” as a random factor, and we compared differences between pairs of sites.

We used network analyses (NA) on the same subset of 17 sharks, on the sharks tagged at Galapagos and Malpelo, and in the sharks tagged at Cocos that made inter-island movement, this to describe the local and global structure of networks constructed from pairwise interactions of connected elements in a graphic format node linked by one or a series of edges, based on study of [37], where nodes represent physical locations or centers of information (acoustic receivers) within their environment. The edges are equally variable and represent physical interactions or the mobility of organisms between fixed locations. The presence at and relative movements between sites were used to build a movement matrix. A graphical method of displaying the inter-relationships between the data in the matrix was performed using the library igraph in R 2.3.1 [50,51].

Diel presence was examined by grouping the detections of all sharks at the Cocos array into hourly bins and analyzing the counts per bin using a Fast Fourier Transformation (FFT, periodogram function, R package TSA). We performed basic circular statistics on the hourly detections to determine spacing and angular concentration using Oriana version 4.02 (Kovach Computing Services).

## Results

### General residence and seasonality

**Fig 2:**
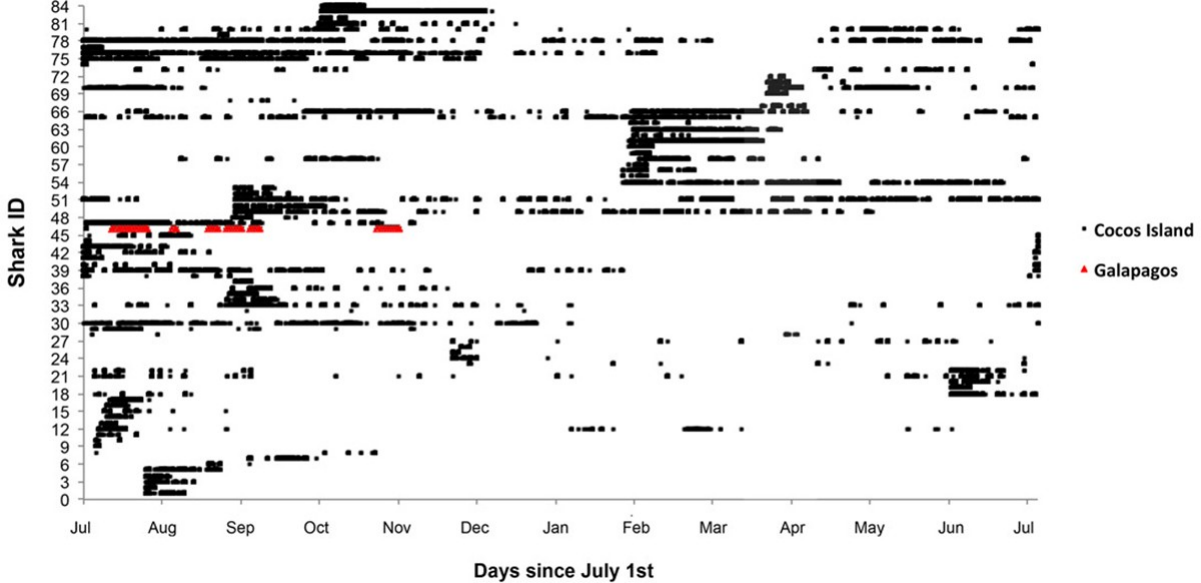
Presence of 84 *S*. *lewini* tagged at Cocos during the study period 2005–2013 (standardized to July 1st). Black squares represent detections in Cocos and the red triangles detections in Galapagos.

A total of 84 *S*. *lewini* were tagged at five locations (Alcyone = 51, Roca Sucia = 16, Manuelita = 12, Dos Amigos = 4, Lobster = 1) between July 2005 and September 2012 (S1 Table). Of these sharks, 62% were female, only a single male was identified, and the gender was not recorded for the remaining 37%. We obtained 184,373 detections that were registered at the six receiver locations around Cocos (Fig 2).

The monitoring duration (defined as the number of days from tagging to the last known detection) ranged from 2–1022 days (median 27.5 d). Eleven sharks were monitored for over a year, although for these sharks the number of days detected at the receivers was low (median 11.5 d). Sharks were absent from the island for extended periods, notably from March through May (Fig 2). Five sharks (#21, 22, 29, 48 and 70) were absent for nine months or longer. Shark #78 was the longest resident to the island, over a period of 197 days from July 2012 to October 2013, with an absence of approximately a month in March 2013.

Eight of the 84 tagged sharks (9.5%) displayed a RI = 1, however with the exception of one individual monitored over 63 consecutive days, their monitoring durations were short (2–11 d). Ten additional sharks with monitoring duration ranging from 120 to 1022 d displayed an RI<0.1. Overall, the RI for hammerheads at Cocos was 0.52±0.31 d SD. If we only consider those sharks with monitoring durations of 120 days or more (N = 27), the RI is 0.24±0.21 d SD (r = 0.44, df = 25, p<0.05).

### Site-specific distribution

**Fig 3:**
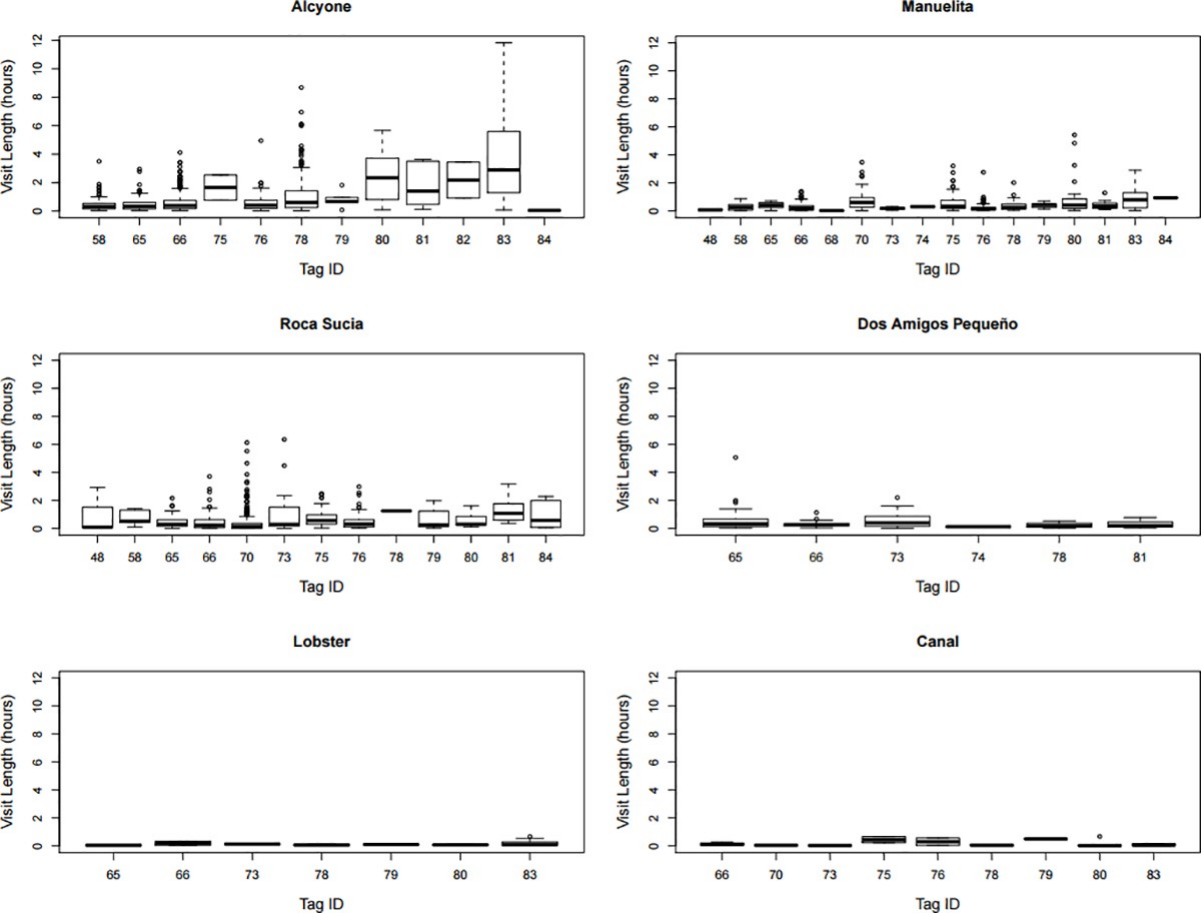
Visit length. Median, quartiles, error bars and outlying points per site from 17 sharks, all of which were being tracked from September 2011 to November 2013.

The comparative use of the six sites was analysed from September 2011 to November 2013, during which period 17 of the sharks were being monitored. Single detections were rare (<0.01% of detections). A total of 2034 residency events were obtained, ranging between individuals from 30 to 387 events, and between locations from 26 events (by 9 individuals at Canal) to 835 events (by 12 individuals at Alcyone). When compared with a null model, our mixed model (Table 1) showed that *Site* was a significant factor determining visit length (χ^2^(5d.f.) = 244.83, p<0.001). Pairwise comparison between sites showed significant differences between all sites except Roca Sucia and Dos Amigos, with the longest visit durations at Alcyone, while Canal and Lobster displayed the shortest (Fig 3).

**Table 1:** Mixed model results for effects of site on visit length for sharks tagged at Cocos Island.

**Random Effects**					
	Variance	Std. Dev.			
Tag ID (intercept)	0.2912	0.5396			
Residual	1.0636	1.0313			
**Fixed Effect**					
	Estimate	St. Error	df	T value	P
Alcyone (Intercept)	1.22804	0.15253	18.9	8.051	<0.001
Canal	-1.46012	0.21117	2022.7	-6.914	<0.001
Dos Amigos	-0.48049	0.12922	1992.4	-3.718	<0.001
Lobster	-2.15042	0.16260	2027.5	-13.225	<0.001
Manuelita	-0.77533	0.07349	2016.1	-10.525	<0.001
Roca Sucia	-0.46899	0.08071	1953.0	-5.811	<0.001

**Fig 4:**
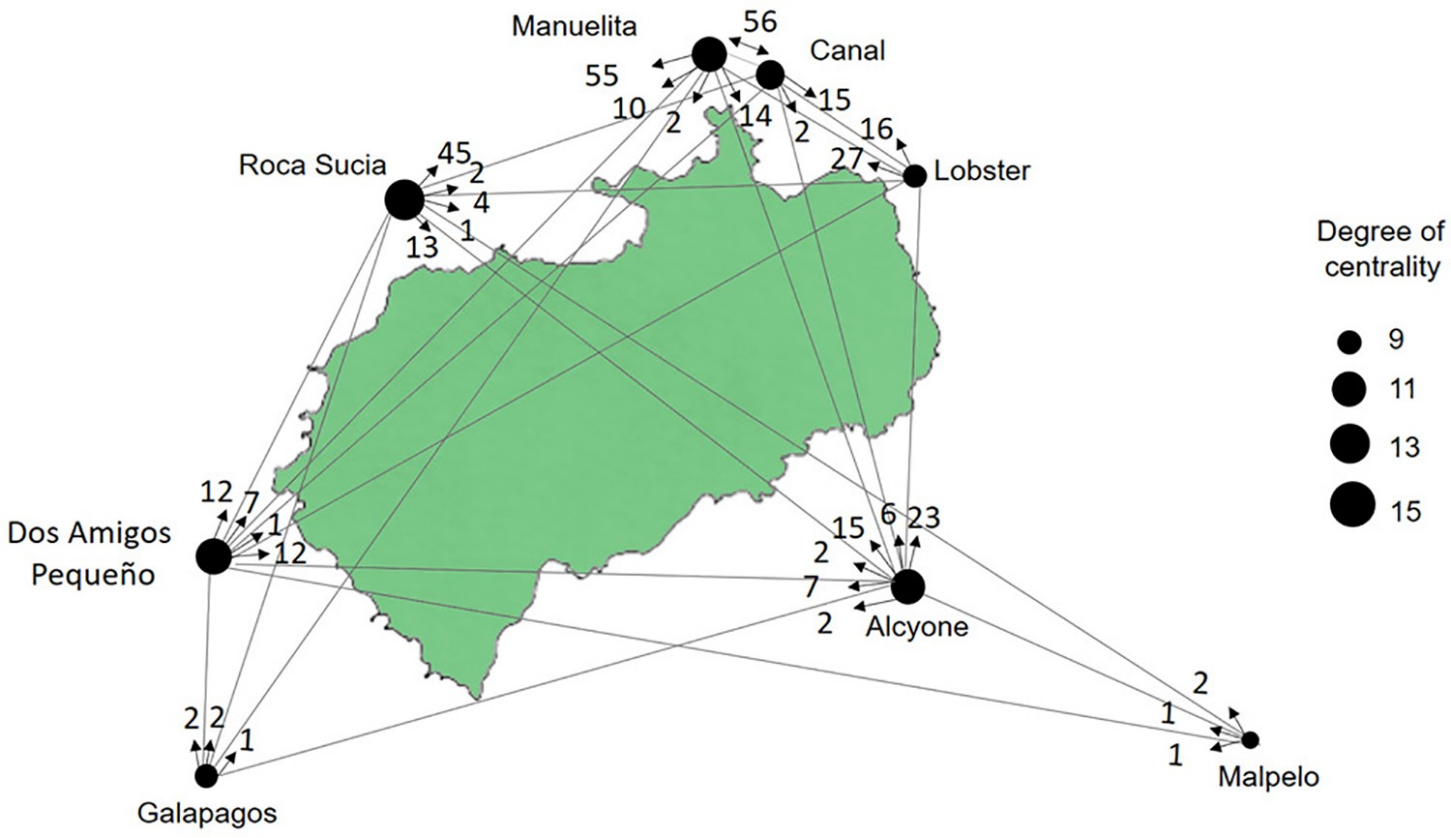
Network analyses (NA) of 17 sharks tagged at Cocos and of 9 sharks tagged at Galapagos and Malpelo detected at Cocos. The edges represent the mobility of the sharks between fixed locations.

Network analysis showed that despite residency being higher at Alcyone, the site with the greatest number of connections and movements was Manuelita, as shown in Fig 4, where the size of the nodes represents the degree, or the probability of a node for being part of the dispersal in the network. The arrows with the numbers represents the direction and the amount of movements between the sites. In contrast, Alcyone and Roca Sucia were the most important locations at which long-distance movements to or from Malpelo and Galapagos were initiated or ended.

### Diel presence at Cocos Island

**Fig 5:**
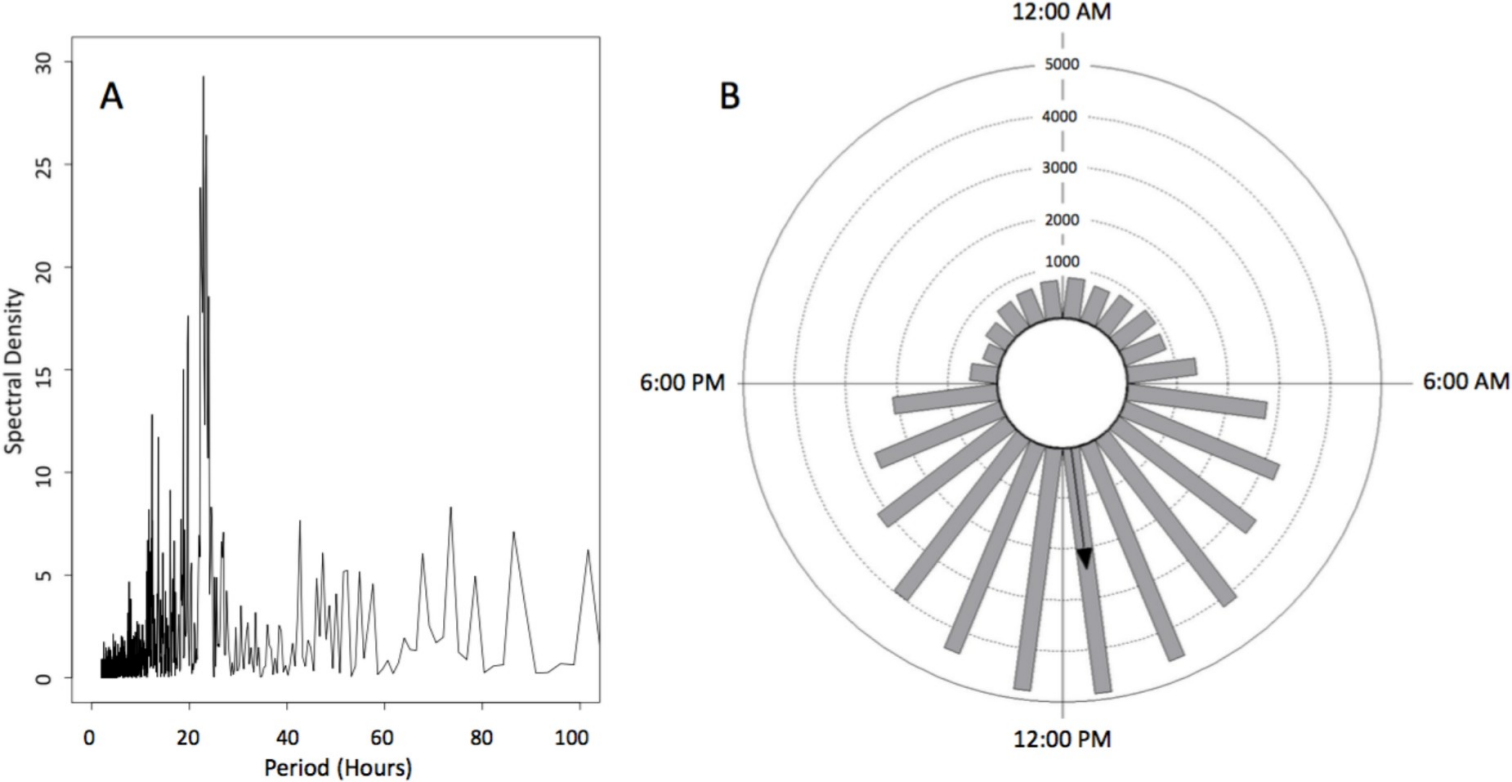
Diel presence of scalloped hammerhead sharks at Cocos. (a) Spectral analysis of the number of tagged sharks present hourly at the Cocos array. (b) basic circular graphic on the hourly detections to determine diel presence.

Spectral analysis of the number of tagged sharks present hourly at the Cocos array showed that there was a strong peak at approximately 24 hours, indicating a diel pattern of presence at the island (Fig 5A). Most of the detections occurred between dawn and dusk (approximately 6 am and 6 pm local time throughout the year). The mean timing of detections was 11:30 am (Rayleigh Test Z = 12499, P <0.01) with an angular concentration (r) of 0.485 (Fig 5B), while Rao’s Spacing Test showed that the timing of detections was non-uniform (U = 186, p<0.01).

### Regional connectivity

**Fig 6:**
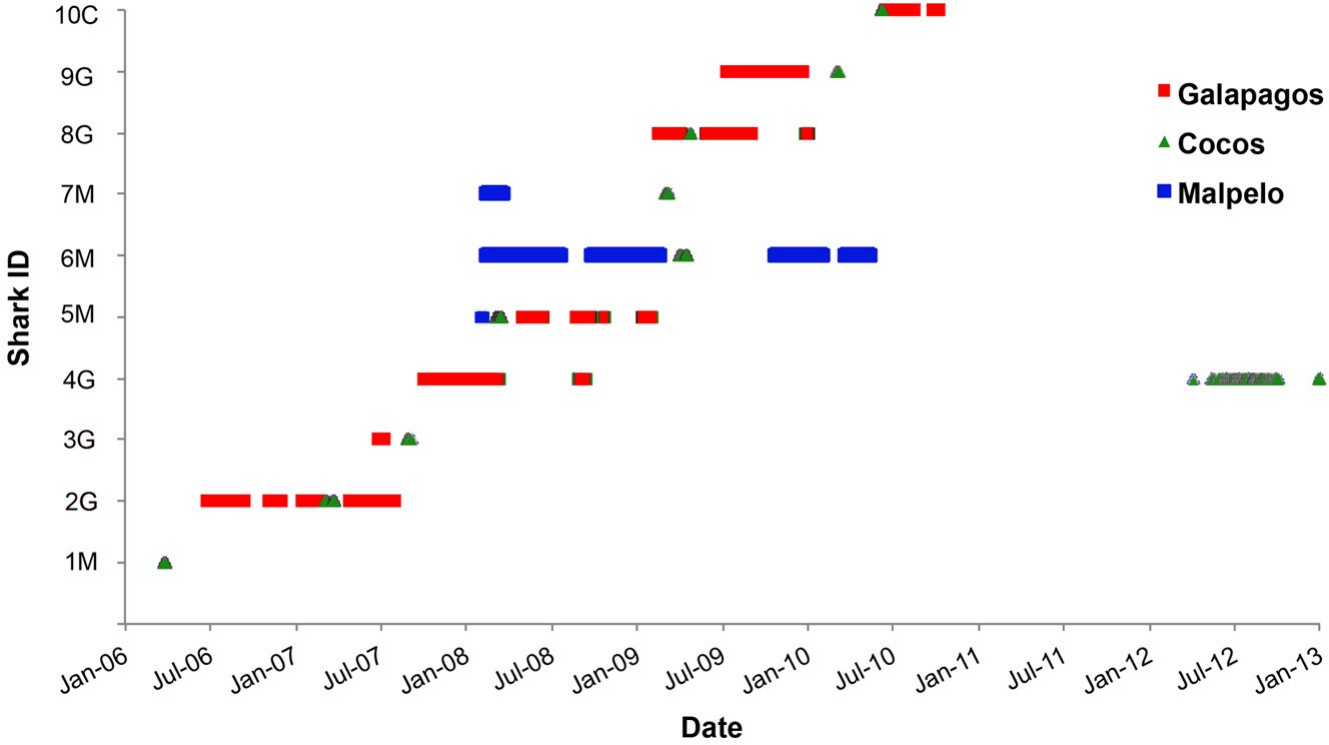
Chronology of scalloped hammerhead sharks tagged at Cocos (C), Galapagos (G) and Malpelo (M), and their detections in the other islands of ETP. Red squares indicate detections in Galapagos, the green triangles the Cocos detections and the blue squares detections in Malpelo.

Ten sharks made inter-island movements in relation to Cocos (Fig 6, S2 Table). However of these, only one individual was tagged at Cocos. In contrast, five sharks (of 210 tagged) moved from Galapagos to Cocos (two of which subsequently returned to Galapagos), and four sharks (of 83 tagged) moved from Malpelo to Cocos. One of the latter (Shark #5M) continued onwards to Galapagos after a brief stay of five days at Cocos. While the dataset is small, it is worth noting that seven of the arrivals at Cocos occurred in April-May, and with one exception (Shark #4G), their visits to the island were brief (1–5 days) and their corresponding residency indices were low (mean RI = 0.014).

The inter-MPA movements made by these sharks were generally relatively fast, indicating direct routes between the islands (Table 2), the exceptions to this being the movement from Cocos to Malpelo, which took over six months, and one shark that was registered at Cocos after four years since its last detection at Galapagos.

**Table 2:** Movements of hammerhead sharks, *S*. *lewini* between the islands of Cocos, Malpelo and Galapagos.

	Cocos to Galapagos	Galapagos to Cocos	Cocos to Malpelo	Malpelo to Cocos
Number of trips	4	5	1	4
Median travel time (days)	33.5	52	189	46
Max travel time (days)	52	1298	189	113
Min travel time (days)	10	15	189	30
Distance (km)	710	710	627	627
Max speed in a straight line (ms^-1^)	0.82	0.55	0.04	0.24

## Discussion

Scalloped hammerhead sharks form large aggregations at several locations around Cocos Island, similar to Galapagos, Malpelo and other oceanic islands in the Eastern Tropical Pacific [17,18,19,24]. The results of our tagging studies suggest that these aggregations are fluid, such that individuals do not remain at the island constantly throughout the year or season, but associate with the island mostly during daytime hours, throughout the year, punctuated by absences. In addition, that sharks should return to Cocos after absences of nine months or greater is worthy of note, and indicates the importance of the island to this species.

Only one male was identified among all sharks tagged. A female-skewed population was similarly observed in the Galapagos Marine Reserve, where only 6 of 71 tagged sharks were male [17]. Sexual segregation in sharks is fairly common, and female *S*. *lewini* are reported to form large aggregations at shallow seamounts and islets, although males are also found within these groups [24]. The authors [52,53] reported that sharks appeared uninterested in feeding when in these aggregations, and suggested that they may serve an energy optimization function or as a landmark location from which to carry out nocturnal foraging activities offshore. They also postulated that an additional benefit of seamount residence may be the ease in which social activities leading to mating could occur. Mating of hammerhead sharks at Cocos Island close to the Manuelita aggregation site was recently recorded by a local dive guide and described [54].

The overall residency index for sharks tagged at Cocos was 0.52±0.31 SD, although this value may be skewed by high values obtained from short monitoring periods and by the limited spatial coverage of our array. A better indication of residency may be that obtained from those sharks whose tracks lasted at least 120 days (N = 27), and whose overall value was 0.24±0.21 d SD. This figure is comparable with values for hammerhead monitoring of similar lengths from Malpelo (RI = 0.33, Bessudo & Soler, Fundación Malpelo, unpublished data) and Galapagos (RI = 0.27) ([17], Galapagos Science Center, unpublished data). This indicates that while they do spend a significant proportion of their time around the island and at the neighbouring Alcyone seamount, their home range extends beyond the limits of the receiver stations, either at other locations close to the island, or offshore. Sharks could be moving towards the chain of seamounts along the Cocos Ridge, which extends to the southwest of the island towards the Galapagos Archipelago, some 690 km away. The Cocos Ridge is a chain of seamounts that extends to the southwest, linking Cocos Island with the Galapagos Archipelago. One of these seamounts, Las Gemelas, located 50 km southwest of Cocos, is well known by fishers as a site of large schools of fish [55], and was included as a No-Take Zone in the recent creation of the Seamounts Marine Management Area in 2013, a nearly 10,000 km^2^ rectangle surrounding Cocos Island National Park’s current MPA. At least one hammerhead was subsequently reported to have made a return movement from Cocos Island to this area in 2016 [56]. Short distance movements (<50 km) such as these were also reported for scalloped hammerhead sharks in Galapagos, where individuals frequently moved between the islands of Darwin and Wolf. These back and forth trips lasted less than 5 days [19]. Many fish tend to reside in particular areas for days, weeks or months, often coinciding with the availability of prey [57], presence of cleaning stations [58], or for reproductive purposes [5,59].

The offshore seamount Alcyone received both the highest number of visits and the visits with the longest duration by the seventeen sharks used to evaluate site preference. It was also the site that provided the greatest overall number of detections (102,911) and the site where the greatest number of tagged sharks were recorded (68 of 84 sharks used in this study). Alcyone is one of the closest seamounts to Cocos Island, located only 3.7 km to the south at a depth of 30 m, compared to Las Gemelas Seamount located 50 km to the southwest at a depth of 180 m. Las Gemelas is one of the summits of the Cocos Ridge, a chain of seamounts that extends to the southwest, linking Cocos Island with the Galapagos Archipelago. Sharks displayed varying degrees of residency at Roca Sucia, Manuelita and Dos Amigos Pequeño as well. Recreational divers have also consistently recorded large numbers of sharks for the past twenty years at all of these sites [23,32]. Permanent cleaning stations exist in Alcyone, Manuelita and Roca Sucia, where scalloped hammerhead sharks are regularly observed swimming through static schools of barberfish (*Johnrandallia nigrirostris*) that feed on their ectoparasites [2,23]. Similar cleaning stations occur at Darwin and Wolf Islands in nearby Galapagos [17] and in Malpelo island (Bessudo & Soler, personal communication). Cleaning stations provide a physical link between the reef environment of the cleaner fish and the pelagic environment of the sharks [8,9], and are often located on shallow seamounts and steep coastal drop-offs. Other large pelagic species also make use of reef fish for cleaning purposes. For example, giant manta rays, *Manta birostris*, were observed being cleaned at Komodo National Park [58] and the Revillagigedo Archipelago by the endemic Clarion Angelfish (Ketchum, personal communication), and the shortfin sunfish, *Mola alexandrini*, may be regularly found being cleaned at a single location at Isabela Island, Galapagos Marine Reserve [60]. Alcyone may also function as a central navigation reference from which the sharks depart each night, presumably to forage in open waters [24,61,6,9]. There was a strong diel signal of the presence of sharks at the island, with sharks moving away from the receiver locations around dusk and returning generally around dawn. Similar diel patterns were observed in El Bajo Espíritu Santo and Galapagos, where sharks that subsequently tracked actively throughout the night moved offshore for distances of several kilometres, presumably to forage [9,17,61].

The nightly excursions of hammerhead sharks residing at oceanic islands could exceed 40 km [9], whereas movements to Las Gemelas seamount occur at a distance of 37 km beyond the current Cocos Island MPA [56], thus implying that sharks at Cocos may be moving in and out of the protected area (22 km radius) on a daily basis. In this case, protection would be limited to the daytime hours spent at the cleaning stations close to the island and not during the night when they move offshore to forage, nor when they move to a nearby seamount outside of the MPA. This increases their vulnerability to longline fishing in the region, which generally operates from dusk to dawn. Moreover, hammerhead sharks are obligate ram ventilators [62], and are therefore highly vulnerable to mortality if unable to swim forward to aerate their gills when caught on a line. Post-release survivorship of sharks that have spent hours on a line may be fairly low [63], and hammerhead sharks exhibit among the highest vulnerabilities to bycatch among shark and ray species [64], the best conservation strategies recommended are those that decrease interactions with fisheries in the first place. Hammerhead abundance at Cocos has declined by 45% over the past two decades [32], probably due to a combination of illegal fishing within and legal fishing beyond the boundaries the Cocos MPA, taking advantage of their movements inside and outside protected waters.

Hammerheads were less abundant at Cocos at the beginning of the year, especially in March [23,32]. This seasonal pattern also occurs at Galapagos [17] and at Malpelo, where [18] reported that many of the sharks that left the island in this period were seemingly pregnant, and speculated that they were migrating to coastal waters to give birth before returning to Malpelo later in the year. Although only a single hammerhead shark tagged at Cocos was later detected at another site within the region (Galapagos), several sharks tagged at Galapagos (N = 5) and Malpelo (N = 4) were detected at Cocos. These inter-MPA movements were generally made over relatively short periods of time, indicating direct routes between the islands (Table 2), whereas movements between the different islands within the Galapagos archipelago often took many weeks [19]. The low residency index at Cocos of the visiting hammerheads during these months suggests that the Galapagos and Malpelo populations may use Cocos as a navigational waypoint or stopover during a seasonal movement to more distant locations, perhaps to coastal waters of Costa Rica, Panama, Colombia and, to a lesser extent, Ecuador, where large numbers of neonate and juvenile *S*. *lewini* are landed by artisanal fisheries [28,65,66,67,68]. Some of the larger coastal lagoons in this region have been identified as nursery habitat [28,69,70]. Although neonate *S*. *lewini* are caught in those areas throughout the year, catches seem to be higher in the months of April and May [28,69,71], coinciding with the period of absence of large adults from oceanic islands. A similar pattern has been observed at the oceanic Revillagigedo Islands, where detections of hammerheads decrease after April (Aldana & Ketchum, unpublished data).

### Implications for management and conservation

Cocos Island is home to aggregations of several shark species, of which *S*. *lewini* is perhaps the most iconic. Individual sharks move between Cocos and other oceanic islands within the ETP. Given the lack of nursery habitat at most of these islands, it is likely that individuals also migrate towards the Pacific coast of Central and South America at certain times of year to give birth [71]. Although these and other sharks are protected inside the marine reserves surrounding the oceanic islands of Galapagos, Cocos and Malpelo, particular life cycle of hammerheads, which includes migratory nature and juvenile habitat, suggests that a more regional approach contemplating protection of their migratory corridors (swimways) and nursery areas is warranted. In August 2013, Ecuador passed legislation banning the retention of hammerheads (*S*. *lewini* and *Sphyrna zygaena*) taken by the industrial fleet, and limiting the retention of incidental catch of hammerheads to five immature individuals (<150 cm total length) per trip, for small scale artisanal fishers (Ministerial Decree 116–2013). In 2005, Costa Rica passed a Fisheries Law, which prohibits landing sharks without their fins naturally attached to their bodies (Ley de Pesca y Acuicultura #8436). However, this law has done little to reduce overfishing of sharks in Costa Rican waters, as it only addresses the practice of finning [34], and does not properly address shark overfishing. In addition, Costa Rica is still struggling to eliminate fishing from Cocos Island National Park [33]. Costa Rica recently expanded protection around Cocos Island by creating the Seamounts Marine Management Area (SMMA), which consists of a 9640 km^2^ rectangle surrounding Cocos Island National Park [72]. Within this area, a no-take zone was designated around a group of seamounts approximately 74 km to the southwest of the island, including Las Gemelas seamount. However, industrial fishing is still permitted within the rest of the SMMA and it is unclear how the area will be enforced. Despite the challenges, the SMMA may serve as a model for larger scale protection of hammerheads and other endangered marine species that migrate between a triangle of MPAs: Galapagos, Malpelo and Cocos.

## Supporting information

S1 Table Sharks tagged at Cocos Island. Scalloped hammerhead sharks tagged at Cocos Island from July 2005 to November 2013 (F: female, M: male, ND: not determined).
(PDF)

S2 Table Scalloped hammerhead sharks tagged at Cocos (C), Malpelo (M) and Galapagos (G). Sharks which are then detected in Cocos and other islands of the ETP during the 2005–2013 study period. (F: female, ND: not determined).
(PDF)

S1 Fig Chronology of each receiver during the study period from July 2005 to November 2013. Dates on which receivers were on place, available to collect data.
(PDF)
