# Supplementary material for: Movements of scalloped hammerhead sharks (Sphyrna lewini) at Cocos Island, Costa Rica and between oceanic islands in the Eastern Tropical Pacific
Source: PLoS One. 2019 Mar 12;14(3):e0213741. doi: 10.1371/journal.pone.0213741 (PMC6413943; doi:10.1371/journal.pone.0213741)
Supplement: S1 Table — Scalloped hammerhead sharks tagged at Cocos Island from July 2005 to November 2013 (F: female, M: male, ND: not determined). (PDF) [file pone.0213741.s001.pdf]

Shark number	Tag ID	Sex	Tagging date	Tagging location	Date last detected	Total detection in Cocos (days)
1	2401	F	25/07/2005	Alcyone	08/08/2005	12
2	2402	F	25/07/2005	Alcyone	28/07/2005	4
3	2403	F	25/07/2005	Alcyone	12/08/2005	14
4	2407	F	25/07/2005	Alcyone	02/08/2005	7
5	2408	F	25/07/2005	Alcyone	22/08/2005	22
6	2405	F	17/08/2005	Roca Sucia	22/08/2005	4
7	2409	F	01/09/2005	Manuelita	24/09/2005	13
8	2410	F	28/09/2005	Manuelita	20/10/2005	6
9	3090	F	06/07/2006	Dos Amigos Pequeño	07/07/2006	2
10	3097	F	06/07/2006	Dos Amigos Pequeño	15/07/2006	2
11	3091	F	07/07/2006	Alcyone	21/07/2006	6
12	3093	F	07/07/2006	Alcyone	21/05/2008	38
13	3092	F	08/07/2006	Roca Sucia	13/07/2006	4
14	3096	F	09/07/2006	Dos Amigos Pequeño	19/07/2006	8
15	3098	F	09/07/2006	Dos Amigos Pequeño	04/08/2006	7
16	3095	F	10/07/2006	Alcyone	17/07/2006	8
17	3099	F	10/07/2006	Roca Sucia	23/07/2006	13
18	4996	ND	28/05/2007	Alcyone	09/08/2007	23
19	4997	ND	28/05/2007	Alcyone	04/06/2007	8
20	4998	ND	28/05/2007	Alcyone	11/06/2007	12
21	4999	ND	28/05/2007	Manuelita	10/05/2009	33
22	5000	ND	28/05/2007	Alcyone	05/04/2009	30
23	5001	ND	31/05/2007	Manuelita	06/04/2008	4
24	5004	F	17/11/2007	Alcyone	28/11/2007	8

	25	5009	F	17/11/2007	Manuelita	21/11/2007	2
	26	5010	F	17/11/2007	Manuelita	25/11/2007	3
	27	5013	F	18/11/2007	Manuelita	29/05/2008	14
	28	13419	F	12/06/2008	Roca Sucia	30/03/2011	7
	29	54440	ND	25/03/2009	Roca Sucia	03/11/2009	89
	30	54441	ND	26/03/2009	Alcyone	01/08/2010	151
	31	56634	ND	26/03/2009	Roca Sucia	03/04/2009	8
	32	56631	ND	27/03/2009	Manuelita	15/12/2009	13
	33	56633	ND	24/08/2009	Roca Sucia	22/11/2010	63
	34	54445	ND	25/08/2009	Alcyone	12/09/2009	13
	35	54443	ND	26/08/2009	Alcyone	05/09/2009	11
	36	54442	ND	27/08/2009	Roca Sucia	12/11/2009	20
	37	54449	ND	28/08/2009	Alcyone	03/09/2009	7
	38	38085	F	27/06/2010	Alcyone	26/08/2010	8
	39	38093	F	27/06/2010	Alcyone	22/01/2011	76
	40	38088	F	29/06/2010	Roca Sucia	03/08/2010	9
	41	38090	F	29/06/2010	Alcyone	08/07/2010	8
	42	38092	ND	29/06/2010	Alcyone	18/08/2010	20
	43	38081	F	30/06/2010	Alcyone	01/08/2010	25
	44	38084	F	30/06/2010	Alcyone	03/07/2010	3
	45	38086	F	30/06/2010	Roca Sucia	10/08/2010	18
	46	38087	F	02/07/2010	Alcyone	29/10/2010	1
	47	38079	F	02/07/2010	Alcyone	04/11/2010	60
*	48	21980	ND	27/08/2010	Roca Sucia	20/09/2011	8
	49	21977	ND	28/08/2010	Alcyone	27/04/2011	97
	50	21978	ND	28/08/2010	Alcyone	01/10/2010	26
	51	21979	ND	28/08/2010	Roca Sucia	21/07/2011	137
	52	21981	ND	28/08/2010	Roca Sucia	17/09/2010	12
	53	21982	ND	28/08/2010	Roca Sucia	11/09/2010	10
	54	32345	F	23/01/2011	Manuelita	16/06/2011	105
	55	32347	F	23/01/2011	Lobster	01/02/2011	6
	56	32358	F	23/01/2011	Manuelita	19/02/2011	20
	57	32342	ND	25/01/2011	Alcyone	01/02/2011	6
*	58	32344	F	25/01/2011	Alcyone	19/10/2011	61
	59	32348	F	25/01/2011	Alcyone	02/02/2011	8

	60	32351	ND	25/01/2011	Alcyone	03/02/2011	9
	61	32352	F	25/01/2011	Alcyone	17/03/2011	51
	62	32357	F	25/01/2011	Alcyone	17/02/2011	10
	63	32361	F	25/01/2011	Alcyone	24/03/2011	48
	64	32349	F	26/01/2011	Alcyone	17/02/2011	8
*	65	32354	ND	26/01/2011	Alcyone	07/03/2012	104
*	66	32360	ND	26/01/2011	Alcyone	01/01/2012	128
	67	42224	ND	17/03/2011	Alcyone	02/04/2011	9
*	68	42226	ND	17/03/2011	Alcyone	17/09/2011	5
	69	42228	ND	18/03/2011	Manuelita	26/03/2011	7
*	70	42956	ND	19/03/2011	Roca Sucia	24/07/2012	70
	71	42223	ND	19/03/2011	Roca Sucia	17/04/2011	9
	72	42955	F	20/03/2011	Alcyone	10/04/2011	4
*	73	42227	F	20/03/2011	Alcyone	09/10/2011	31
*	74	28551	F	27/06/2012	Manuelita	02/07/2012	4
*	75	28549	M	28/06/2012	Alcyone	27/11/2012	75
*	76	14033	F	30/06/2012	Alcyone	04/02/2013	152
	77	28547	F	30/06/2012	Alcyone	08/07/2012	8
*	78	14031	F	01/07/2012	Alcyone	25/10/2013	197
*	79	3440	F	22/08/2012	Alcyone	25/08/2012	4
*	80	21291	F	29/09/2012	Alcyone	01/11/2013	66
*	81	15268	F	29/09/2012	Alcyone	04/12/2012	26
*	82	21289	F	30/09/2012	Alcyone	09/10/2012	7
*	83	21290	F	30/09/2012	Alcyone	01/12/2012	63
*	84	15267	F	30/09/2012	Alcyone	16/10/2012	16

(*) sharks used in the residency index and network analyses
