# Supplementary material for: Movements of scalloped hammerhead sharks (Sphyrna lewini) at Cocos Island, Costa Rica and between oceanic islands in the Eastern Tropical Pacific
Source: PLoS One. 2019 Mar 12;14(3):e0213741. doi: 10.1371/journal.pone.0213741 (PMC6413943; doi:10.1371/journal.pone.0213741)
Supplement: S2 Table — Sharks which are then detected in Cocos and other islands of the ETP during the 2005–2013 study period. (F: female, ND: not determined). (PDF) [file pone.0213741.s002.pdf]

Shark ID	Tag ID	Sex	Tagging date	Date last detected	Total detection period (days)	Total detection in Cocos (days)	Residence index
1M	134	ND	01/03/2006	20/04/2006	51	1	0.020
2G	1206	F	21/07/2006	25/08/2007	401	2	0.005
3G	5625	F	23/07/2007	05/10/2007	75	3	0.040
4G	7289	F	31/10/2007	25/01/2013	1914	67	0.035
5M	6962	F	03/03/2008	24/02/2009	359	5	0.014
6M	6937	ND	09/03/2008	06/06/2010	820	3	0.004
7M	6928	ND	10/03/2008	01/04/2009	388	3	0.008
8G	52956	F	15/03/2009	28/01/2010	320	1	0.003
9G	55940	F	06/08/2009	01/04/2010	239	2	0.008
10C	38087	F	02/07/2010	29/10/2010	120	1	0.008
