# Supplementary figures and images for: Movements of scalloped hammerhead sharks (Sphyrna lewini) at Cocos Island, Costa Rica and between oceanic islands in the Eastern Tropical Pacific
Source: PLoS One. 2019 Mar 12;14(3):e0213741. doi: 10.1371/journal.pone.0213741 (PMC6413943; doi:10.1371/journal.pone.0213741)

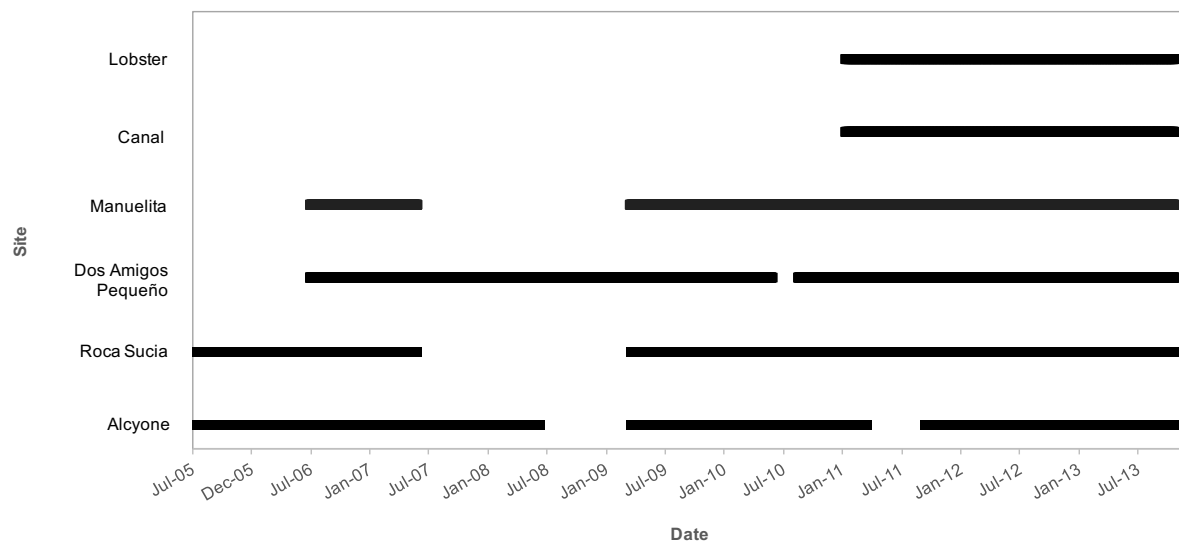

Supplement: S1 Fig — Dates on which receivers were on place, available to collect data. (PDF) [file pone.0213741.s003.pdf]
